# Improving “Life Chances”: Surveying the Anti-Transgender Backlash, and Offering a Transgender Equity Impact Assessment Tool for Policy Analysis

**DOI:** 10.1017/jme.2022.89

**Published:** 2022

**Authors:** M. Killian Kinney, Taylor E. Pearson, Julie Ralston Aoki

**Affiliations:** 1: PACIFIC UNIVERSITY, FOREST GROVE, OR, USA; 2: INDEPENDENT ATTORNEY/SOCIAL WORKER; 3: MITCHELL HAMLINE SCHOOL OF LAW, ST PAUL, MN, USA.

**Keywords:** Transgender Rights, Policy Analysis, Social Determinants of Health, Social Justice

## Abstract

Transgender inclusion within policy is critical yet often missing. We propose a policy tool to assesses human rights, access to resources and opportunities, language, and implications for transgender and nonbinary individuals. Acknowledging trans communities as standard policy practice can serve as an essential practice to shift dialogue and norms.

*“Trans people are told by the law, state agencies, private discriminators and our families that we are impossible people who cannot exist, cannot be seen, cannot be classified, and cannot fit anywhere.”*

- Dean Spade^1^

*“You have to act as if it were possible to radically transform the world. And you have to do it all the time.”*

- Angela Davis

## I. Introduction

A significant legislative struggle over transgender equity currently exists in America. Anti-trans legislation across the U.S. has increased in record amounts, from 79 bills in 2020 to 147 bills in 2021, with 2022 already showing signs of reaching a new historical high.^2^ In January, the Human Rights Campaign has warned that the 2022 legislative session could host an “intentional, coordinated attack” on transgender individuals and particularly youth.^3^ Within the first week of 2022 alone seven states proposed anti-trans bills, including bills restricting access to sports, gender-affirming care, and bathrooms.^4^ That number has skyrocketed to 30 states at the time this article was being written (see Figure 1).^5^ However, so too have bills to protect the rights of trans and nonbinary youth and adults, such as inclusive nondiscrimination policy and gender marker and name change on state identification cards. The transgender equity impact assessment tool is a critical addition to tools for analyzing potential impacts of proposed and existing legislation and educating policymakers and others about transgender issues.

In general, many people, including many policymakers, lack understanding about gender diverse people and how policy impacts their health and wellbeing. In brief, gender diverse people are anyone who does not identify as cisgender — or with a gender that aligns with the sex they were assigned at birth, which includes binary trans individuals (trans women and trans men) and trans nonbinary individuals (anyone who does not identify as exclusively a woman or man). Diversity of gender identity and expression is expansive, and present across cultures and throughout history.^6^ According to current national estimates, between 0.6% and 5.0% of adults in the United States claim a gender identity under the broad transgender category.^7^ Trans and nonbinary individuals are each unique though they often share similar experiences of discrimination and stigma.^8^ Understanding the nuances of gendered experiences by trans and nonbinary people are best assisted by people within these communities.

Building from the growing number of racial equity and healthy equity impact assessment tools for assessing policy, we propose a transgender equity impact assessment tool designed for use by policymakers, advocates, and community members to assess proposed and existing legislation for gender inclusivity and discrimination.

Despite a changing social climate of acceptance and some progress in trans advocacy,^9^ discriminatory legislation continues to be proposed and passed with negative repercussions for transgender and nonbinary (T&N) communities. Numerous bills each year are proposed that explicitly attack the rights of transgender people.^10^ The recent waves of bills appear to be motivated by animus against trans people. In these situations, education and equity impact assessment processes are not likely to change proponents’ minds, although they may still create a platform for reaching voters who may be unaware and/or open to change. Other policies are less obviously harmful; these require a critical lens and awareness of intersectional identities to understand their impacts, which an equity impact assessment process can provide. Further, similar to the recognition that addressing structural racism requires both the dismantling of structurally racist systems *and* forward-looking action focused on healing and repairing,^11^ gender equity work must be ongoing, focusing both on preventing and undoing harmful policies as well as on the promotion of gender-affirming and inclusive policies that protect the human rights of T&N people while also recognizing the unique needs and assets of people of all genders. Policymakers, government officials, advocates, and community members are increasingly recognizing the value and importance of equity impact assessment tools for this kind of work, particularly in the context of advancing racial equity and health equity.^12^ At the heart of the transgender equity impact assessment tool is community engagement through a community advisory board that is constantly working and ready to analyze policy when needed. This approach is an ongoing commitment *with* and *by* community for T&N inclusion and equity.

Building from the growing number of racial equity and healthy equity impact assessment tools for assessing policy,^13^ we propose a transgender equity impact assessment tool designed for use by policymakers, advocates, and community members to assess proposed and existing legislation for gender inclusivity and discrimination. In Section II, we explain the policy landscape of discriminatory and affirming legislation relating to T&N and Lesbian, Gay, Bisexual, Transgender, Queer (LGBTQ) identity or status. In Section III, we provide an overview of the research documenting the adverse health impacts of discrimination on T&N people, demonstrating the importance of inclusive policy to address health inequities. In Section IV, we explain the tool, the theory and values that inform it, and describe how it can be applied. Finally, in Section V, we conclude by describing our goals for future transparent and gender-affirming policy work.

### A. Positionality Statement^14^

For readers to understand the perspective of the authors, we offer a positionality statement for transparency of our identities and perspectives. The first author is a queer, nonbinary, white, currently nondisabled assistant professor. They have worked with transgender and nonbinary people for eight years, including healthcare, policy analysis, advocacy, and research to improve the lives of gender diverse people. As a trans person, I prioritize work that assists policymakers to leverage their positions to improve the lives of gender diverse people and to effectively combat harmful anti-trans policies. The second author is a cisgender, demisexual, white, able-bodied woman who is an emerging lawyer and social worker specializing in civil rights and public policy with particular interest in critical social theories as applied to gender equity. The third author is a bi-racial (Japanese/white), cisgender, heterosexual, nondisabled woman who has lived with both racism and privilege. The authorship team was intentional to represent multiple different roles, identities, and histories. Anti-oppression and activism for social justice are shared values among the authors that propelled the development of this transgender equity impact assessment tool.

## II. The Legal Landscape

The struggle for equitable and just treatment of T&N people is one of the defining issues of our time. It is happening across our communities in hospitals and doctors’ offices, schools, libraries, workplaces, places of worship, senior living centers, prisons, and other places that impact the full spectrum of human experience. This struggle is shaped by policy decisions made by local, state, federal, and Tribal governments,^15^ as well as by court cases challenging or interpreting these policy actions.

The public policy pendulum swings radically and erratically. In June 2020, for example, the Trump administration reintroduced measures to roll back certain Patient Protection and Affordable Care Act (“ACA”) restrictions on discrimination against T&N patients in the provision of health care and health insurance.^16^ Just days later, the Supreme Court ruled that terminating an individual’s employment on the basis of their gender or sexual orientation is a violation of Title VII the Civil Rights Act of 1964.^17^ In 2021, the U.S. Supreme Court let stand a federal court of appeals decision holding that a school board’s policy of requiring transgender students to use bathrooms separate from other students violated federal laws against sex discrimination in education.^18^ In that same month, the Court issued a decision that the City of Philadelphia improperly terminated a contract with Catholic Social Services for refusing to allow married LGBTQ couples to be considered for foster parents, even though this violated the city’s antidiscrimination laws.^19^

Unfortunately, the pendulum all too often swings against the side of equity and nondiscrimination. In 2021, state legislatures considered a staggering 150 anti-trans and anti-LGBTQ bills, at least 15 of which were ultimately signed into law.^20^ Anti-trans and anti-LGBTQ policies impact a wide and deep range of human needs and activities, including access to essential healthcare services; access to public accommodations such as public restrooms; access to human services, such as eligibility to serve as foster or adoptive parents; access to supportive educational environments, including school athletics; and the right to indicate on driver’s licenses, birth certificates, death certificates, and other official government records a person’s correct gender identity.

The struggle for human rights and civil rights protections for T&N people spans many years and is happening across all types of and at all levels of government. For this article, however, we chose to focus primarily on state legislative bills introduced and/or adopted during the 2021 legislative session. We focus on state bills because actions by state legislatures can have powerful upward and downward effects, significantly influencing the national and local policy landscapes in both positive and negative ways. Sometimes, states have been more progressive on social justice issues, including gender diversity, creating space for municipal governments within those states to also be progressive, and helping to drive changing social norms in ways that lead to positive federal policy change.^21^ More recently, local governments have been the ones to introduce progressive measures, leading some states to react with preemptive laws to strip or limit local authority to enact T&N affirming measures, even when no municipality within the specific state had introduced such a measure.^22^ We chose 2021 because it is a recent year, and because the plethora of state bills that were introduced that year, mostly anti-trans and anti-LGBTQ but also some affirming and inclusive bills, provide ample illustration of the wide and deep impact of such policies.

### A. Anti-Trans Legislation

Anti-trans legislation affects all areas of life for T&N people. Below, we call out recent examples from the health care sector, public accommodations, and school athletic programs.

Arkansas recently passed a bill prohibiting health care professionals from providing gender-affirming health services such as hormone therapy or plastic surgery to minors, and it also bars insurance companies from reimbursing or otherwise covering such services.^23^ The state has further prohibited as discriminatory “tak[ing] an adverse action against, or communicat[ing] a threat of adverse action to” health care professionals or institutions who refuse to provide such services on the basis of conscientious objection — despite the fact that federal courts have held that Section 1557 of the ACA’s prohibition of sex-based discrimination includes gender identity and applies to all health care programs receiving federal funding.^24^ Shortly following the passage of the bill, the US District Court in the Eastern District of Arkansas granted a preliminary injunction which kept the law from being implemented, though that decision is under appeal in the Eighth Circuit Court of Appeals..^25^

Tennessee enacted two of the handful of “bathroom bills” that surface each year.^26^ One, an addition to the state’s building regulations code, requires all businesses allowing trans patrons to use the bathrooms of their choice to post brightly-colored signage reading “THIS FACILITY MAINTAINS A POLICY OF ALLOWING THE USE OF RESTROOMS BY EITHER BIOLOGICAL SEX, REGARDLESS OF THE DESIGNATION ON THE RESTROOM.”^27^ In the same year, Tennessee also passed the “Accommodations for All Children Act,” a law paradoxically requiring public schools to provide “reasonable accommodations” upon request to students and employees using multi-occupancy bathrooms and changing rooms but simultaneously prohibiting them from allowing individuals to use facilities designated for the opposite sex.^28^ The business signage case has been permanently enjoined after a Tennessee judge ruled “It would do a disservice to the First Amendment to judge the Act for anything other than what it is: a brazen attempt to single out trans-inclusive establishments and force them to parrot a message that they reasonably believe would sow fear and misunderstanding about the very transgender Tennesseans whom those establishments are trying to provide with some semblance of a safe and welcoming environment. The Act fails the constitutional standard that actually applies to it, and the inquiry should end there.”^29^ Though the school bathroom bill faced a court challenge as well, it is still in effect after the two students on behalf of whom the case was brought, moved out of state citing the hostile environment at the schools.^30^ Eight laws curtailing trans athletes’ ability to participate in sports intended for their gender went into effect in seven different states in 2021 alone, many of them incorporating virtually identical language:^31^ public-school-sponsored intramural and interscholastic athletic teams and clubs must be designated as male, female, or coed, with membership to be determined solely according to “biological sex” as assigned at birth.^32^ Proponents of these laws argue that certain gender equality and trans-affirming policies weigh in favor of such exclusions.

### B. Trans-Affirming Legislation

During the 2021 legislative session, fewer states introduced trans- and LGBTQ-affirming bills, but there were some small successes. In 2021, the American Civil Liberties Union (“ACLU”) identified 40 “good” LGBTQ equality bills across; unsurprisingly, none of these bills were introduced in any of the states that proposed overtly exclusionary policies.^33^ Nevertheless, five of these 40 bills were enacted into law. We highlight examples relating to health care, and government administration and data collection.

Unlike Arkansas’ laws permitting health care insurers to refuse to cover gender-affirming services on the basis of conscience,^34^ Washington state’s “Gender Affirming Treatment Act” prohibits insurers from denying or limiting coverage of medically necessary treatments prescribed “because of, related to, or consistent with a person’s gender expression or identity” as well as from imposing “blanket exclusions” on such treatments.^35^ The law further delineates types of patients for whom these treatments may be prescribed, including “two spirit, … nonbinary, intersex, and other gender diverse individuals,” in addition to transgender individuals,^36^ thus explicitly creating space for patients who have traditionally been excluded from the health care debate.^37^ California passed three positive laws in 2021 concerning public administration, specifically the modification of birth certificates,^38^ the completion of death certificates,^39^ and the establishment of a pilot data collection program focusing on gender identity and sexual orientation.^40^ Under these new laws, a person can request, the issuance of a new birth certificate reflective of their gender identity (including an option for nonbinary), such that their “legal gender” conforms with their gender identity.^41^ Additionally, California death certificates are to be issued with the decedents’ reported gender identities rather than biological sex, unless other identifying documents specify differently.^42^ Finally, the California State Department of Health now collects and tracks these data points for all suicides and homicides in six pilot counties “to encourage a better understanding of disparities in mortality in rate in the LGBTQ community” and support responses to such disparities.^43^

### C. Summary Thoughts

The importance of the policy debates and decisions happening in our state legislatures over civil rights and basic human rights for transgender and nonbinary people cannot be overstated. These state actions not only directly impact people’s lives, health, and welfare of T&N people, but they also shape our vision of ourselves as a society and a larger community. In other words, they impact T&N people by shaping the contexts within which they receive services and engage with their communities and, also, by creating an overarching social landscape within which anti-trans sentiment and rhetoric cultivate misunderstanding of, hostility towards, and even violence against trans folks.^44^ One parent of a trans youth expressed, “I have nightmares about [my daughter’s] future already… . I already have obstacles in place because she’s trans. We weren’t exactly excited to find out that she was trans -- it’s scary. I lay in bed and think about whether she’ll get a job one day or whether she’ll find a partner, or will she be murdered by somebody simply for existing.”^45^ Trans rights have become a politicized topic with T&N people as pawns and recipients of mounting hostility rather than simply about access to fundamental health and other human needs.^46^ For T&N people, social justice includes health equity, legal representation, and social inclusion, among others. In addition to codified human rights, shifting social norms and lowering stigma can be achieved by policy that is explicitly antidiscriminatory and intended to protect and expand resources and opportunities.^47^ Dean Spade argues for radical legal reform for trans liberation and justice that includes examining the role of policies in trans peoples’ lives, particularly those policies that create conditions that diminish their “life chances” and shorten their lives.^48^

Civil rights will not be enough; we need social rights as well. Gender-based oppression is not only or primarily accomplished through the power of the states: police, courts, and laws… To make it possible for people to transcend gender lines, we must not only change laws and policies, we need to change social attitudes and raise awareness of gender harassment.^49^

This symbolic value is particularly important, given that these debates and decisions are occurring in an increasingly polarized sociopolitical environment.^50^

## III. Why Transgender and Nonbinary Gender Affirming and Inclusive Policy Matters

Harmful policies explicitly and implicitly impact the lives of T&N people. The language and punishments proposed in bills intended to limit the rights and resources of gender diverse people create a hostile environment — even when they do not pass. They convey the message that trans people are perceived as a threat and invalid. Having what Dean Spade calls “life chances”^51^ up for debate is corrosive to T&N wellbeing. Public policy is a social determinant of health and wellbeing,^52^ and it shapes people’s experiences of other social determinants of health (SDOH). For example, laws prohibiting discrimination in schools and by employers implicate all of the other SDOH: educational access, community context, access to housing, economic stability, and access to healthcare.^53^ As such, policies have individual, organizational, and social implications that must be considered. Next, we will discuss direct and indirect implications for T&N people at each level.

### A. Implications for Individuals and Public Health

Consistent with previous stigma research, T&N individuals often report that they are concerned with the lack of protective policies that are inclusive of their gender identity and expression, especially when this absence is used to delegitimize their gender and claim to basic human rights.^54^ Stigma experienced by T&N people, due to discrimination on the basis of their socially nonconforming gender identities, contributes to increased depression, anxiety, and suicidality.^55^ In particular, misgendering (especially among those who use they/them pronouns) has been found to be positively associated with psychological distress.^56^ Further, a lack of protective policies can contribute to internalized stigma (e.g., inferiority) and increased discrimination, while inclusive protective policies are more likely to promote equity and invoke community belonging.^57^ Despite research indicating a need for interventions to *support and protect* T&N youth to reduce the risk of adverse mental health outcomes, the opposite is happening and instead, these youth are the ongoing targets of anti-trans bills. The deleterious effect of these bills on their mental health and wellbeing is a public health concern. The 2021 National Survey on LGBTQ Youth Mental Health found disproportionately high rates of discrimination and concerning mental health outcomes, and it connected these with issues such as conversion therapy and lack of access to gender marker changes, which are also subjects of current policy initiatives.^58^ T&N youth reported experiencing symptoms of generalized anxiety disorder (77%) and major depressive disorder (70%). Even more dire, 52% of T&N youth had seriously considered attempting suicide and 20% had attempted suicide in the last year. Nearly a quarter of transgender and nonbinary youth (24%) had experienced discrimination based on their sexual orientation or gender identity in the past year, and the accumulation of multiple types of discrimination was positively correlated with attempted suicide in the last year. Those who were subjected to conversion therapy reported more than twice the rate of attempting suicide (19%) compared to cisgender LGBQ youth who were not (9%). This startling data illustrates the harm of conversation therapy, and it is consistent with the condemnation of this practice by professional mental health associations.^59^ This is why advocates seek formal bans of practices to change one’s sexuality or gender identity.

Another protective policy that influences health outcomes for youth is the ability to change legal documents (e.g., name and gender markers on driver’s licenses and birth certificates), which is associated with lower rates of suicide attempts. Higher rates of suicidality among T&N youth have been attributed to lack of support, social stigma, and internalized transphobia related to their gender nonconformity.^60^ Unsurprisingly, T&N adults also experience adverse mental health impacts from discrimination and stigma; for instance, 41% of respondents to the National Transgender Discrimination Survey reported attempting suicide, compared with 1.6% in the general population.^61^ Historically, bathrooms have been a battleground of human rights movements for Black people and other people of color, women, people with disabilities, and now for transgender people.^62^ Though the majority of anti-trans bathroom bills have not passed, the repeated proposals and heated discourse around bathroom bills are harmful to T&N people. Bathroom bills place T&N people at risk of violence from verbal and physical assault and mental health risks as a result of facing daily suspicion, harassment, and hostility.^63^ In a study by the UCLA’s Williams Institute, nearly 70% of transgender participants reported experiencing discrimination when trying to use gendered public restrooms.^64^ According to the 2015 U.S. Transgender Survey (USTS), bathrooms are increasingly dangerous spaces for transgender persons at school, work, and other public locations.^65^ The majority (59.0%) of respondents reported avoidance of bathrooms for fear of harassment or other problems, of which 89% reported “holding it,” 52% reported limiting fluid and food intake to limit necessary bathroom use, and 12.0% reported urinary tract infections or related infections as a result. In school, transgender students reported significantly less perceived safety than their cisgender counterparts, and that their relationships between gender and school safety was significantly mediated by feeling safe to use the bathroom.^66^ Further, being denied access to bathrooms has been linked to reduced mental health and increased suicidality among T&N college students.^67^ It has been established that minority stressors^68^ have a cumulative effect on mental and physical health,^69^ including diminishing psychological wellbeing.^70^ Further, chronic exposure to stressors in people’s residential, occupational, and other environments can have a biological impact on them, which has been called “biological weathering.”^71^ In other words, “stress-mediated wear and tear on the body” can contribute to health disparities.^72^ These findings have been extensively studied among Black and Indigenous populations, and these studies have exposed the connection of marginalization associated with breast cancer, chronic inflammation, accelerated aging, and intergenerational trauma.^73^ Though LGBTQ research is just beginning to explore the mental health and biological effects of marginalization, the existing literature shows more anxiety among children with gender dysphoria,^74^ more reported health-related problems among LGBTQ individuals who experience greater levels of microaggressions,^75^ and correlations between LGBTQ-based victimization and high risk for depression and PTSD symptoms..^76^ A study of 65 healthy transmen showed elevated diurnal cortisol levels throughout the day due to transitioning identity stress related to public restrooms, which particularly highlights the direct connection with bills about bathroom usage.^77^ The given chronic experiences of transphobia and discrimination among T&N individuals and the accumulative biological weathering support an argument for policy as a social determinant of health.

### B. Organizational Implications

Educational and healthcare settings represent two indicators of poor social determinants of health.^78^ Minority stressors^79^ (e.g., gender-based microaggressions, aggressions, and discrimination) contribute to reduced health and wellbeing, including internalized transphobia.^80^ Unlike other life arenas, educational and healthcare settings are necessities in people’s lives and are critical areas for T&N protections and disruption of harmful policy and practice.

Though the hostility of school environments for T&N youth have been well established,^81^ and this hostility has been found to severely compromise the psychosocial wellbeing of LGBTQ youth,^82^ research has also found that inclusive school policies can *protect* against adverse mental health outcomes.^83^ The 2019 National School Climate Survey found that the majority of students (79.1%) reported any form of anti-bullying policy at their school, only 12.5% of T&N students reported that their school had a policy or guidelines regarding T&N students.^84^ When a school had a *comprehensive* harassment policy (compared to a generic policy that did not include sexual orientation or gender identity and expression), LGBTQ students were less likely to hear negative remarks about gender expression and transgender people, more likely to report incidents, and school staff were more likely to intervene. Research has found that some teachers report not knowing how to affirmingly engage with gender minority students (e.g., bathroom usage, pronouns),^85^ indicating a need for ongoing gender-inclusive training. When gender-inclusive policies were present, T&N students reported better experiences with name and pronoun usage, access to bathrooms and locker rooms that aligned with their gender, and gender expression (no gendered dress codes).^86^ T&N students in schools with such policies were less likely to miss school and reported a greater sense of belonging to their school community.^87^ T&N people are a medically underserved population^88^ who face pervasive discrimination in healthcare access.^89^ Healthcare settings are sites of systemic microaggressions, both when seeking urgent care in emergency rooms and gender-affirming care from primary care providers.^90^ “It is frustrating, but it’s also definitely invalidating because you have to sit through a very uncomfortable situation anyway because nobody really wants to be at the doctor and misgendered, it’s like I’m already not feeling well, you have to kick me while I’m down, too?”^91^ T&N individuals continue to be invisible in or ignored by health care systems through informational erasure (e.g., unprepared health providers) and institutional erasure (e.g., lack of trans-inclusive forms and policies).^92^ For example, when insurance policies for transitional surgeries are written exclusively for transitioning from male to female or female to male, a claim from a nonbinary person could be denied. Primary barriers to accessing gender-affirming health care include difficulty finding a trans-affirming provider or a provider who offers services related to medical transitioning, verbal mistreatment (i.e., abusive language) or physical mistreatment (i.e., rough handling) within healthcare settings (including by provider), and denial of care (by provider and insurance).^93^ Access to healthcare is also intertwined with employment discrimination and health insurance, considering T&N high rates of unemployment (15%; three times the national average), lack of insurance (14%), and living in poverty (29%).^94^ As a result of healthcare mistreatment, some T&N people seek gender-related clinics and professionals, which can be limited in some geographical areas.^95^ Despite progress, such as the World Professional Association of Transgender Health (WPATH) Standards of Care that establish guidelines for practitioners to provide gender-affirming care,^96^ T&N individuals are 2.34 times more likely to be denied care across their lifetime compared to their cisgender LGB counterparts.^97^ T&N adults who have experienced discrimination from medical professionals may withhold information or postpone care,^98^ and fear of negative experiences has led some T&N individuals to self-treat^99^ or to pass as cisgender to avoid discrimination.^100^ Another important consideration is that health care practitioners report feeling ill-equipped to serve the T&N patients.^101^ As such, “The existence of an actual trans person within systems such as health care is too often unanticipated and produces a social emergency of sorts because both staff and systems are unprepared for this reality.”.”^102^ Conversely, when T&N adults had gender-affirming primary care physicians, they were eight times more likely to have pursued a medical intervention than those without.^103^ Professional organizations also can influence policy and social change by taking a stand against anti-trans policies and advocating for protections. Similar to how the mental health associations collectively stood up against conversion therapy, medical associations (i.e., American Academy of Pediatrics,^104^ Pediatric Endocrine Society^105^) have publicly opposed anti-trans bills that attack trans rights. The American Medical Association called anti-trans bills that could prohibit access to gender-affirming transitional care for minors “a dangerous intrusion into the practice of medicine” and could have “tragic health consequences, both mental and physical.”^106^

### C. Systemic and Societal Implications

In addition to the importance of understanding individual and interpersonal stigma, structural stigma is also critical to the health and wellbeing of T&N people.^107^ Structural stigma helps explain why some individuals flourish and why others do not.^108^ Policy, and legislative policy in particular, can perpetuate and entrench stigma and discrimination, which causes adverse health impacts, or it can support and protect people.^109^ Exclusionary or harmful policies are forms of structural stigma that uphold health inequities and perpetuate social stigma. For example, religious exemptions policies have created legal exemptions for discrimination, such as doctors refusing transition-related care to T&N patients,^110^ which perpetuate the acceptability of restricting and denying health care and services to T&N people.^111^ Additionally, hostile public discourse around trans-related policies (e.g., bathroom bills) magnify trans-invalidating hate speech and heighten safety concerns, the impact of which has yet to be studied but is acutely felt by T&N individuals.

Cisnormative assumptions in policy reinforce oppression systemically and organizationally and require change at the same systemic levels.^112^ To mitigate these adverse outcomes, antidiscrimination legislation and other protective policies must encompass sexual orientation, gender identity and expression.^113^ While sexual orientation has been accepted for inclusion in protective policy, protections for gender identity and expression has been met with greater resistance. In particular, nonbinary inclusion in non-discrimination policy has been debated.^114^ Some have argued for policy and research explicitly intended to reduce the structural stigma associated with marginalization.^115^ With this approach, policy could become transparent about intended public health impacts while also educating the public; this could help highlight the reciprocal responsibility between policy and societal norms.^116^

In response to the political climate towards T&N people, we propose a transgender equity impact assessment tool for policy analysis. The tool is designed to be used in three primary ways: 1) to support systematic critique of anti-trans policies and illuminate their negative health and discriminatory impacts; 2) to aid the development of affirming and equitable policies for T&N communities, especially by those who are not familiar with T&N issues; and 3) to raise public awareness and advance T&N affirming advocacy.

## IV. The Transgender Equity Impact Assessment Tool for Policy Analysis

In response to the political climate towards T&N people, we propose a transgender equity impact assessment tool for policy analysis. The tool is designed to be used in three primary ways: 1) to support systematic critique of anti-trans policies and illuminate their negative health and discriminatory impacts; 2) to aid the development of affirming and equitable policies for T&N communities, especially by those who are not familiar with T&N issues; and 3) to raise public awareness and advance T&N affirming advocacy. The transgender equity impact assessment tool provides a systematic way of analyzing policy for key factors that impacts trans lives. Though it is not a comprehensive evaluation tool, the six steps of the transgender equity impact assessment tool provide a framework that can be expanded upon and informed by current equitable conditions (see Figure 2).

### Step #1: Engage Community Members

Before analyzing any policy, it is important to create a diverse, engaged, and ongoing community advisory board (CAB) in the state or locality wishing to implement the equity tool. The CABs should encompass T&N community stakeholders, including community leaders, students, and families. At times, it may be appropriate to bring in additional community members on a policy-by-policy basis (i.e., T&N youth to analyze high school sports regulations for transgender athletes). Community advisory boards (CAB) are a common form of community engagement.^117^ Utilizing CABs in policy work can increase trust with communities and increase the likelihood that policy will lead to improved outcomes for community members. For example, the inclusion of CABs in sugary drink tax policies are considered a best practice for ensuring sustained public support for these policies and that tax revenues are distributed in ways that will benefit community members who are most marginalized by structural health inequities, addressing concerns about tax regressivity.^118^ In the gender inclusivity context, when unsure about appropriate affirming language and if a policy could be misinterpreted, a CAB can offer insight about language and lived-experience regarding how policy has been leveraged to assist and hinder T&N individuals.

Concerns about community involvement relate to meaningful engagement on the part of the organizers — concerns including co-optation,^119^ tokenization,^120^ or even merely listening without appropriately responding. Instead, effective community advisory boards require clear parameters in terms of time commitment, roles (educate? make policy recommendations?), and decision-making power.^121^ A CAB should have authority to make decisions, shape policy, and in some cases (such as for state or local agency policy) set policy; otherwise, members can become frustrated if their advice is disregarded or if they feel they have no influence over policy outcomes and are just “window dressing.”^122^ Similarly, compensation for time is essential to an effective CAB, particularly one composed of marginalized populations who may be experiencing financial and other hardships.^123^ When honored as direct connections to community knowledge, CABs can be effective tools for community-responsive policy, trouble-shooting, and community buy-in.^124^ Community engagement in policy making can be empowering for community members and can contribute to a new social construct “in which society places greater trust in — and empowers — the public to play a far more active role in the functioning of their government…there is inherent value in rejuvenating civil society and shifting the focus away from unsustainable entitlements to personal responsibility and solidarity.”^125^ Community engagement is essential to effective and equitable policy development and thus is central to the transgender equity impact assessment tool.

There are many approaches to creating a CAB.^126^ Once created, a CAB can be used on short notice, allowing for a quick and effective response. It is worth reiterating that compensation for time and expertise is an ethical necessity, particularly when considering the emotional labor as well as the value of time being asked of T&N people.^127^

ways a CAB can be helpful:^128^

- Creating a new policy.
- Reviewing drafts of a policy for appropriate language and content.
- Illuminating less obvious harms or negative unintended impacts to T&N people of potential or existing policies.
- Surveillance of proposed policies and enforcement of existing policies, to inform policymakers, other decision-makers, and advocates about both affirming and harmful policies.

A CAB also offers a dual direction of communication that can build trust with communities and provide a direct line of response to community members when issues arise in their communities.

In addition to community members, consultation with researchers, educators, medical and mental health providers, and organizational leadership with work or life experience with the community can provide valuable expert perspectives. Additionally, such professionals may provide expert testimony or perspectives when such is needed. Policymakers could benefit from having ready access to a network of experts across topic areas. However, professional experts cannot replace community experts.

### Step #2: Assess for Human Rights

Once a policy has been identified, the first consideration is whether it is protecting or threatening the human rights of T&N people. This step of analysis looks for explicit and implicit purposes of the policy. Begin with the following prompts:

- What is known about this policy and topic in the context of T&N people?
  - Have similar versions been proposed before? If so, what was said about them?
- Who is proposing this policy and what is their history on T&N and other civil rights issues?
  - What other types of bills or policies have the lead authors or proponents supported or opposed in the past?
  - What is the stated intent of the policy?
  - What groups support or oppose this kind of policy??
- Does the policy have *explicit* or *coded* (implicit) gendered language which is likely to impact T&N individuals?
- What, if any, T&N human rights are explicitly identified in this policy?
- If not explicit, what T&N human rights arguably could be impacted by this policy, and in what ways?

When assessing language for explicit and implicit impact on T&N people, a CAB has an essential role in this work. A policy might not appear on its face to impact T&N individuals. However, critical assessment of the language by community members can reveal implicit impacts on T&N people. One such example is a policy on school sports that does not explicitly mention transgender athletes.^129^ However, codifying access to sports only according to biological sex is an indirect way of discriminating against T&N individuals.

If the policy is found to be protective of T&N human rights, continue through the steps to identify supporting evidence about what and how rights are protected with particular attention to step #4. If the policy threatens human rights, the subsequent steps will help to substantiate the harm to T&N individuals. Many policies will not explicitly identify their benefits or harms; thus, the following steps will help to identify more nuanced impacts of policy on T&N individuals.

### Step #3: Assess the Impact on T&N People’s Ability to Access Resources and Opportunities or “Life Chances”

After the human rights framing of the policy has been assessed, Step #3 explores deeper implications of the policy for T&N individuals. Key areas of access include public accommodations, housing, education, employment, and gender-affirming health care, including puberty blockers, hormone therapy, and surgeries.

Again thinking about the explicit and implicit intent of the policy, answer the following prompts.

- Will this policy increase access to basic needs (bathrooms, shelter, safety, education)?
- Does this policy explicitly allow T&N people access to opportunities or resources (i.e., adoption, marriage, service in the military) that are allowed to cisgender people?

If access is increased, the analysis in Step #4 will help to identify if language is affirming to all gender diverse people and areas for possible improvements. If access is explicitly hindered or prevented, identify the barriers and their underlying rationale to help with developing counterarguments. If the policy creates obscured or implicit barriers, identify those barriers to make them visible, drawing upon research as much as possible, and explain how this policy can be used to limit access for T&N individuals. If the policy neither increases nor decreases access to life chances, ask whether it can be amended to increase equity for T&N people? For example, if the policy is about the expansion of Medicaid coverage, can trans-affirming care be added?

### Step #4: Assess the Language

Language is neither innocent nor neutral^131^ and carries important social norms and meanings, such as rules, privileges, and punishments.^132^ Similarly, policy language shapes social and cultural norms and understandings. It also can provide clear indicators of the policy’s intended goal, as well as the motivations and mindsets of the policy’s authors and supporters. Courts rely on the specific words in a statute or ordinance to interpret laws and understand legislative intent, so policymakers know that policy language matters. Thus, the specific words used in a policy can have far-reaching implications that should be given appropriate consideration.

It is less likely that T&N community members would recommend outdated, offensive language, which is often seen among policies written by those outside of the affected populations. Whether the policy is likely to have explicit or implicit harmful or positive impacts on T&N people, the words in the policy should be assessed for the following:

- Does the policy use outdated language?
  - If yes, note this as another substantiating argument for not supporting the policy.
- If the policy is otherwise protective and expands life chances, how could the language be changed to improve this policy?
- Is the language used to affirm and be inclusive of gender diverse persons? (i.e., culturally appropriate language used correctly)
  - If not, can the language be changed?
- Does the policy language affirm nonbinary/gender-expansive people (i.e., it does not refer to people exclusively as being “man” or “woman,” and also includes people who are gender fluid, agender)?
  - If not, can the language be changed to be expressly inclusive of people of nonbinary genders?
- Could the language reasonably be used directly or indirectly (through interpretation) to support gender-based exclusion?
- Does the policy use words that have a specialized legal meaning or significance that might not be apparent on its face? This may be an area where help from an expert or legal technical assistance provider may be needed to identify such language.

As a caveat, language is a social construct that is perpetually evolving and changing. With this understanding, the most current best practices in equitable language guided by T&N community members should be used.

### Step #5: Assess Application in Practice

The purpose of this step is to assess the policy’s practical application. This analysis is a culmination of explicit and implicit rights, access, and language that could directly and indirectly impact T&N community members.

- Does the policy language clearly describe what it requires or prohibits, and how it is to be implemented? Vagueness and lack of clarity are a sign of a poorly-drafted policy, and reason enough to discard or not support any policy. Vague laws are vulnerable to legal challenge. Moreover, if a policy is unclear in such a way that it could be interpreted to limit or deny the rights of T&N individuals, it is particularly dangerous.
- How could the policy be interpreted?

Some well-intended policies that appear to be inclusive might not be as inclusive in practice. For example, some organizational bathroom policies have used language such as “a person can use the bathroom that aligns with their gender.” This might appear to be an affirming policy; however, in practice, this policy can still be exclusionary to nonbinary people if only binary gender (men and women) bathrooms are available. A final consideration is whether or not the policy consciously contributes to progressive social change. Ultimately, political work should move our society towards a better world for all of us.

### Step #6: Create a Transgender Equity Impact Assessment Statement

The final stage of the tool is to produce a transgender equity impact assessment statement (TEIAS), drawing from the research, discussion, and analyses conducted as part of the previous steps. Recommended substantive content for a TEIAS includes:^132^

- Purpose of the TEIAS (could be brief boilerplate statements).
- Policy synopsis (could be copied from the policy for transparency, if a synopsis is available and is accurately written).
- Transgender equity considerations (summarizes human rights protections or restrictions, access to resources and opportunities, based upon input from a CAB, professional experts, and research).
- Anticipated impacts (based on input from a CAB, professional experts, and research, including possible (mis)interpretation and enforcement). This section provides crucial information for raising public awareness about the experiences and existence of T&N people and how the policy (and others like it) are likely to impact their health and lives.
- Recommendations (based on input from a CAB, professional experts, and research).
- Transgender equity impact assessment statements can be used to:
- Publicly disseminate information about the harmful mental and physical health impacts of discriminatory and unfair policies for T&N people and communities in general
- Raise awareness about systemic transphobia, discrimination, and unjust and unfair treatment of T&N people
- Garner support and advance advocacy for affirming and inclusive legislation for T&N people
- Share across the aisles to recruit political co-authors and supporters
- Create a repository of information and research that can be used in other jurisdictions when similar policies are proposed or being analyzed

Box 1 A Truncated Example Of a Current Policy

**Policy:** Participation in School Sports (Indiana HB1041)

**Purpose of the Transgender Equity Impact Assessment Statement (TEIAS):** The TEIAS is a proactive tool to evaluate policies and the anticipated impact on T&N individuals in a collaborative decision-making process with the community…

**Purpose of IN HB1041:** To restrict participation in school sports to same-sex teams according to assigned sex at birth or coeducational/mixed teams, which would implicitly ban transgender youth from participating in school sports.

**HB1041 Synopsis:** “Participation in school sports. Requires, for purposes of interscholastic athletic events, school corporations, public schools, nonpublic schools, and certain athletic associations to expressly designate an athletic team or sport as one of the following: (1) A male, men’s, or boys’ team or sport. (2) A female, women’s, or girls’ team or sport. (3) A coeducational or mixed team or sport. Prohibits a male, based on the student’s biological sex at birth in accordance with the student’s genetics and reproductive biology, from participating on an athletic team or sport designated as being a female, women’s, or girls’ athletic team or sport.”

**Transgender (In)Equity:** HB1041 impinges on the rights of T&N athletes to participate in sports teams that correspond with their gender by requiring binary sex-based teams. These restrictions force nonbinary individuals who do not identify as exclusively male or female and binary transgender individuals (trans boys and trans girls) to either not participate in school sports or to be part of a sports team that does not match their gender. It specifically targets trans girls for exclusion. HB1041 also would force T&N students to out themselves publicly and creates a high risk of emotional and physical abuse for them.

**Anticipated Impact:** If passed, HB1041 would perpetuate stigma and exclusion of T&N youth as they navigate significant social and emotional development stages. Dr. J. D. Fortenberry, the founder of Indiana’s only adolescent gender health program, testified against anti-trans sports bans, speaking about the adverse social, emotional, and biological impact of such discrimination and exclusion. T&N youth face higher rates of depression, anxiety, and suicidality due to exclusion and stigma. In response to HB1041, S. Ames, the director of advocacy and government affairs for the Trevor Project, stated “‘[Indiana’s] bill claimed to solve a problem of ‘fairness’ in school sports…that didn’t exist, but its negative impacts on the mental health and well-being of trans and nonbinary youth — young people who already face disproportionate rates of bullying, depression, and suicide — are very real.’”^133^ In other words, HB1041 would only exacerbate these risk factors for T&N youth contributing to an already significant and well-documented public health concern.

**Recommendations:** Based on the literature, expert advice, and the community advisory board, HB1041 has been identified as a policy harmful to T&N youth. The ACLU has recommended contacting your local representative to ask them to vote no on HB1041…

**Policy Supporters:** Rep. Michelle Davis (author), Rep. Chris Jeter (co-author), Rep. Joanna King (co-author), Rep. Robert Heaton (co-author), and Sen. Stacey Donato (sponsor).

**Policy location:** http://iga.in.gov/legislative/2022/bills/house/1041#document-8c4d8ab1

**Contributors:** M. K. Kinney, T. E. Pearson, and J. Ralston Aoki

A primary strength of the TEIAS is that they could raise awareness in a concise and easily digestible format for most laypersons, as well as help advocates in multiple jurisdictions who seek to oppose harmful policies and advance inclusive, affirming policies. Similar to the growing wave of racial equity impact assessment tools, as impact statements such as this become used more frequently and, eventually, are normalized as part of policy development processes, this will increase public understanding of T&N human rights and public policy issues.^134^

### D. Who Should Use the Transgender Equity Impact Assessment Tool?

The transgender equity impact assessment tool is intended to be used by anyone interested in analyzing policy for T&N inclusion and raising awareness about factors impacting T&N communities. The level of comprehensibility for the tool makes it appropriate and useful for a wide range of professional, educational, and lay applications. Legal scholars or others doing policy analysis and/or concerned legislators can use the tool to evaluate policies that exist or are being proposed for their impacts on T&N communities. Strategic policy analysis and decision-making could be shared with CAB members and expert council. Additionally, the use of the TEIAS models transparency in political exchange. Within educational settings, the tool provides a concrete practice tool that could be used in policy, advocacy, and diversity courses (i.e., law, public health, social work, sociology, women’s and gender studies) to understand the implications of policy on T&N lives. For example, an assignment can be structured for individuals or groups of students to complete the tool for a specific policy of interest. Laypersons, including concerned citizens, allies, community members, and activists, can use the tool for critiquing policies anytime.

The TEIAS tool can be used across policies — whether they are gender-affirming, ambiguous impact, or explicitly harmful — to raise awareness of both benefits and harm. In particular, the tool can be used with openly discriminatory anti-trans legislation to critique the policy and raise public awareness for those who may not understand the harm to T&N people. Similarly, the impact statement can be used as a Zap^135^ to disseminate critical information about a harmful policy to the public and hold lawmakers and organizations accountable for their policy actions. The tool also stresses the existence and benefit of CABs and their role in policy work. Ultimately, as users become more experienced with the tool, they are likely to become better at identifying areas for improvement in policy and gaps in practice/protections for proposing new policy.

For jurisdictions or organizations that already use a racial equity or health equity impact assessment or similar tool, specific questions from the TEIAS could be added (rather than using a separate TEIA) to ensure that T&N issues are being addressed. The intersectional nature of human identity should be a foundational consideration of all equity tools. Given the current political climate, the transgender equity impact assessment tool is timely in serving community advocates, educators, and policymakers as they seek to promote policies that positively impact trans communities.

### E. Case Study

To demonstrate the effectiveness of this tool in practice, we now apply it to the Arkansas “Gender Integrity Reinforcement Legislation for Sports (GIRLS) Act,”^136^ initially proposed by the state’s Attorney General as “a preemptive effort” to curtail a then-recent executive order she feared would interfere with children’s, especially young girls’, ability to “‘compete on a level playing field.’”^137^ The law defines “sex” to be immutable and determined by a person’s anatomy at birth. It then requires any school (from elementary to postsecondary) located in Arkansas that receives state funds, and any Arkansas schools that play against these schools in interscholastic, intercollegiate, intramural or club sports, to designate their teams as being specifically for men or boys, for women or girls, or “coed or mixed.” People who are of the “male sex” are prohibited from playing on teams or sports that are designated for “females.” Any school that “knowingly” violates this can be sued by the Attorney General and subject to whatever legal relief is allowed by law, as well as being barred from receiving funds from “any public source.”^138^ The legislators responsible for this law invoked the rationale behind Title IX protections and statements by the late Justice Ginsburg to justify the restrictions imposed: “‘[i]nherent differences’ between men and women … remain cause for celebration, but not for the denigration of the members of either sex or for artificial constraints on an individual’s opportunity.”^139^ Because girls’ and women’s athletic opportunities are still limited, the legislators reasoned,^140^ and because boys and men have “physical and hormonal advantages” over their female counterparts,^141^ girls’ sports need to be insulated from such unfair advantages to maintain equality between the sexes.

Despite this attempt to leverage a seminal decision in equal rights jurisprudence to its advantage; however, the GIRLS Act nevertheless fails at every step of the analytical framework established by the tool we propose here. A staunch Republican with ties to the National Rifle Association, pro-life movement, and Federalist Society,^142^ Arkansas Attorney General Leslie Rutledge is a proven conservative dedicated to “‘fighting the woke agenda of [the] liberal left.’”^143^ These values are shared by many of the legislators who co-sponsored this law after Rutledge first introduced it, including the primary author Senator Missy Irvin, who has been recognized for her efforts “supporting persons with mental illness [and] substance abuse disorders” and founded the Human Rights for Kids Organization.^144^ Even so, the GIRLS Act makes no mention of T&N people’s rights, or of human rights at all, instead focusing entirely on what it characterizes as equal opportunities for young girls.

Assuming arguendo that the law’s purpose is to “promote equality in sports and access to athletic opportunities for girls and women,” the analysis required by a TEIA highlights a critical gap that undermines this goal: The law sets no explicit restrictions on whether girls and women can participate in boys’ or men’s sports.^145^ Instead, it only expressly forbids AMAB people^146^ from participating on female-only sports teams,^147^ implying a similar restriction on AFAB people^148^ participating on male-only sports teams but excluding nonbinary individuals altogether. These omissions carry significant misogynistic, paternalistic, and transphobic implications, and suffer from fundamental flaws in the act’s logic. In other words, the policy argues for the explicit protection of (AFAB) girls and women, but does not mention any parallel protections for (AMAB) boys and men, elucidating the proponents do not view trans women as women nor trans men as men. Girls, by any definition, are erased from the narrative: trans girls cannot play, and trans boys are not worth mentioning at all. Additionally, the law does not offer any reasons why this protection is needed other than merely stating that athletic opportunities for girls and women are still limited compared to those available to their male counterparts. By neglecting to acknowledge deeply rooted systems of structural sexism, particularly gender stereotypes about athleticism and the systemic underfunding of women’s sports, the GIRLS Act fails to justify itself.

Additionally, the act leaves no room for nonbinary athletes to participate in either boys’ or girls’ sports; even “[c]oed or mixed” activities are designated as such based on their participants’ “immutable biological sex as objectively determined by anatomy and genetics existing at the time of birth,”^149^ such that athletes can be either male or female, but nothing else. In requiring athletic programs to be designated based solely on participants’ biological sex, the GIRLS Act forces nonbinary individuals to choose a gender, thus negating the experiences of the nonbinary community and reinforcing the very same archaic gender and sex stereotypes that have so severely limited women’s opportunities in the first place.

Finally, the law effectively perpetuates the same inequalities it purports to resolve by establishing a cause of action against programs found to be in violation. By stripping covered entities that knowingly violate the law of all funding “from any public source,” the GIRLS Act sets schools up to be completely divested of the resources they need to address the “lingering disparities” the Arkansas legislature set out to eliminate, a counterproductive means of enforcement that ultimately defeats the law’s initial purpose.^150^

### F. Limitations of the Tool

Some limitations exist with the use of the transgender equity impact assessment tool. First, the tool is a subjective assessment that may be hindered by one’s implicit biases, for which reason we recommend using the tool in diverse teams, such as CABs. The tool is not comprehensive of all facets of risk and benefits to T&N individuals and communities. Additionally, (mis)interpretation is difficult to anticipate (e.g., Can this be interpreted to be used for gender-based exclusion?). Another important limitation is the lack of publicly available, easily accessible data about T&N populations, which is needed to understand the physical and mental health impacts of policies. T&N people are understudied; and because they make up a small percentage of the population (although likely to be underreported due to stigma and discrimination), studies are difficult to implement and need creative approaches and flexible funders. The limited available data is often behind paywalls,^151^ inhibiting easy and affordable access for CABs, policymakers, and advocates. Further, interpreting and applying social science/public health data requires some specialized knowledge and familiarity with this kind of data, research methods, and often some kind of formal training, which expertise people using the tool for assessments may not possess. Lastly, the time frames of the legislative process can be very quick [typically, anywhere from a week to a month (which would be considered a long time) to develop and write an impact note]. This restricts the available time for research, CAB meetings, and other preparation for using the tool. When possible, standing ad hoc CABs and people with topical expertise can help to resolve some of these time limitations. In other words, rather than using the tool in response to a specific policy, the transgender equity impact assessment tool and CABs can be a process to begin ensuring the ongoing analysis of bills, education, coalition building, and advocacy. The initial time is an investment but will allow for a smooth and quick response, such as is currently needed.

### G. Strengths of the Tool

The transgender equity impact assessment tool can be used for ongoing strategic policy analysis and decision-making that is affirming of T&N people and communities. The development of the tool was informed by the Universal Declaration of Human Rights^152^ and the 2015 USTS,^153^ which identifies risk factors for T&N people. Further, the design of the tool is modeled on racial equity tools that provided structured strategies and products (e.g., impact statements) for policy analysis. Specific examples include the Montgomery County, MD Racial Equity Impact Assessment Tool^154^ and the Racial Equity and Social Justice (RESJ) Impact Statement.^155^ The transgender equity impact assessment tool can be used at any level, including organizational, municipal, state, and federal. For Tribal communities and organizations, further and additional thinking by people grounded in Indigenous lived experiences would be important to ensure that any assessment tool aligns with the specific culture and values of the Tribe or Tribal organization.^156^ The possibility exists for the tool to change and grow to become increasingly comprehensive or to be tailored for specific policy topics, such as healthcare policies. Though the tool can be used in direct response to a particular bill, the greatest benefit of the tool is as an ongoing way of examining policy in communities — especially in the current environment.

## V. Conclusion

In this paper, we presented a brief overview of the legal landscape of bills impacting T&N people. We made an argument for the adverse impact of discriminatory policies and the need for inclusive protective policies to address health inequities. We presented the transgender equity impact assessment tool as an instrument for raising public awareness about the impact of policy on T&N people. We conclude this paper with our goal for the future of policy work.

It is our hope that this paper and the transgender equity impact assessment tool create movement towards a future of policy work characterized by transparency and collaboration to improve the lives of all citizens, including transgender and nonbinary people. Policymakers have an ethical responsibility to conduct their work with intentional awareness and responsibility for the implications of those bills.^157^ Anti-trans policies perpetuate transphobia and incite gender-based discrimination, which have no place in public policy. We hope the transgender impact assessment tool is used to facilitate discussion and hopefully lead to better public policy decisions and transparency in policy work. As equity impact statements become normalized, the goal is to cease the proliferation of anti-trans policies. By centering community in policy work, unintentional harm can be prevented and intentional harm can be faced with the collective power of the people. It cannot be overemphasized that community engagement is integral to policy work.

From classrooms to boardrooms, from reservations to city streets, transcending narrow gender norms can get you harassed, assaulted, or killed. Change won’t come quickly; this struggle is just beginning … this is a movement whose time has come. Join us … Get involved. Because gender rights are human rights, and the time for them is now.^158^

The current environment with increasing anti-trans bills has been described as “very dark, and there’s a strong sense among trans people that we are having the door slammed in our face just as we got our foot in the door;” however, T&N people have been urged to “stand up and fight with every breath that we have.”^159^ And we encourage you to join us.

## Note

The authors have no conflicts to disclose.

## References

[1] D. Spade , Normal Life: Administrative Violence, Critical Trans Politics, and the Limits of Law (Durham: Duke University Press, 2015): 19.

[2] P.M. Bailey, “Exclusive: 2022 Could be Most Anti-Trans Legislative Years in History, Report Says,” *USA Today*, *available at* <https://www.usatoday.com/story/news/2022/01/20/2022-anti-trans-legislation/6571819001/> (January 20, 2022).

[3] *Id.*

[4] M. Lavietes, “At Least 7 States Proposed Anti-Trans Bills in First Week of 2022,” NBC News, January 7, 2022, *available at* <https://www.nbcnews.com/nbc-out/out-politics-and-policy/least-7-states-proposed-anti-trans-bills-first-week-2022-rcna11205> (last visited July 26, 2022).

[5] The map shows states that have proposed anti-trans bills with one (lightest red) to as many as 14 states (dark red, e.g., Tennessee). “Legislative Tracker: Anti-Transgender Legislation,” Freedom for All Americans, *available at* <https://freedomforallamericans.org/legislative-tracker/anti-transgender-legislation/> (last visited June 30, 2022).

[6] American Psychological Association, *Report of the APA Task Force on Gender Identity and Gender Violence* (Washington, DC: American Psychological Association, 2009): at 38; L. Feinberg , Transgender Warriors: Making History from Joan of Arc to Dennis Rodman (Boston: Beacon Press, 1996); H. Frohard-Dourlent, B.A. Clark, M. Doull, and E.M. Saewyc, ‘“I Would Have Preferred More Options’: Accounting for Non-Binary Youth in Health Research,” *Nursing Inquiry* 24, no. 1 (2016): e12150; B. Vincent and A. Manzano, “History and Cultural Diversity,” in C. Richards, W.P. Bouman, and M.-J. Barker, eds., *Genderqueer and Non-Binary Genders* (London: Palgrave Macmillan, 2017): at 11-30.

[7] J.L. Herman , A.R. Flores , T.N.T. Brown , B.D.M. Wilson , and K.J. Conron , *Age of Individuals Who Identify As Transgender in the United States*, Williams Institute Report (January 2017); *Transgender Issues: A Fact Sheet*, Transgender Law and Policy Institute, *available at* <http://www.transgenderlaw.org/resources/transfactsheet.pdf> (last visited June 30, 2022).

[8] M.K. Kinney , Learning to Thrive in a Binary World: Understanding the Gendered Experiences of Nonbinary Individuals and Ways to Bolster Wellbeing (Indianapolis: Indiana University, 2021); I.H. Meyer and D.M. Frost, “Minority Stress and the Health of Sexual Minorities,” in C.J. Patterson and A.R. D’Augelli, eds., *Handbook of Psychology and Sexual Orientation* (New York: Oxford University Press, 2013): 252-266.

[9] *Bostock v. Clayton County*, Georgia, 140 S.Ct. 1731, 1731 (2020); S. Mukherjee, “What the Supreme Court’s Landmark Ruling on LGBTQ Job Discrimination Means for Transgender Health Protections,” *Fortune*, June 15, 2020, *available at* <https://fortune.com/2020/06/15/supreme-court-lgbtq-discrimination-ruling-vote-decision-scotus-transgender-health-care-protections-obama-trump-law-gorsuch/> (last visited July 26, 2022).

[10] M. Lavietes and E. Ramos, “Nearly 240 anti-LGBTQ bills filed in 2022 so far, most of them targeting trans people,” NBC News, March 20, 2022, *available at* <https://www.nbcnews.com/nbc-out/out-politics-and-policy/nearly-240-anti-lgbtq-bills-filed-2022-far-targeting-trans-people-rcna20418> (last visited July 26, 2022).

[11] R. Yearby , C.N. Lewis , K.L. Gilbert , and K. Banks , “Racism Is a Public Health Crisis. Here’s how to respond,” *Data for Progress Report*, September 2020.

[12] Many racial equity assessment tools and processes have been developed, geared towards community organizations, non-profit governance, municipal governments, state and local government agency or department operations, state legislative processes, and so on. See, e.g., *Racial Equity Tools*, *available at* <https://www.racialequitytools.org/> (last visited June 30, 2022); National Association of Chronic Disease Directors, “Moving to Institutional Equity: A Tool to Address Racial Equity for Public Health Practitioners,” *available at* <https://chronicdisease.org/moving-to-institutional-equity-a-tool-to-address-racial-equity-in-public-health/> (last visited June 30, 2022); Local and Regional Government Alliance on Race & Equity, *Racial Equity Toolkit: An Opportunity to Operationalize Equity*, *available at* <https://racialequityalliance.org/wp-content/uploads/2015/10/GARE-Racial_Equity_Toolkit.pdf> (last visited June 30, 2022); Milwaukee, Wisc., Code § 108.03(2)(e) (2021); Council Office of Racial Equity, *available at* <https://www.dcracialequity.org/> (last visited June 30, 2022); Montgomery Cnty., Md., B. 27-19 (2019) (establishing a requirement for new legislation to go through a racial equity and social justice impact assessment process); Wash Rev. Code § 43.20.285 (2022) (establishing a health impact review process for legislation); Iowa Stat. § 2.56 (requiring new criminal law legislation to undergo limited review for impact on “minorities”).

[13] *Id.*

[14] Positionality statements are increasingly used in publications to briefly describe one’s insider/outsider position and the advantages and disadvantages of these positions as situated with the work. One’s positions and worldview can potentially have an impact on their work, including race, gender, sexuality, (dis)ability/chronic illness status, social class, religious/spiritual beliefs, ethics, political values, among others. S.L. Budge , S.L. Katz-Wise , E.N. Tebbe , K.A.S. Howard , C.L. Schneider , and A. Rodriguez , “Transgender Emotional and Coping Processes: Facilitative and Avoidant Coping Throughout Gender Transitioning,” The Counseling Psychologist 41, no. 4 (2013): 601–647; A.G.D. Holmes, “Researcher Positionality – A Consideration of Its Influence and Place in Qualitative Research – A New Researcher Guide,” *International Journal of Education* 8, no. 4 (2020): 1-10, at 1-3; D. Qin, “Positionality,” in N.A. Naples, R.C. Hoogland, M. Wickramasinghe, W. Ching, and A. Wong, eds., *The Wiley Blackwell Encyclopedia of Gender and Sexuality Studies* (Malden: Wiley-Blackwell, 2016); J. Wellington, A.-M. Bathmaker, C. Hunt, G. McCulloch, and P. Sikes, *Succeeding with Your Doctorate* (London: Sage Publications, 2005); P. Greenbank, “The Role of Values in Educational Research: The Case for Reflexivity,” *British Educational Research Journal* 29, no. 6 (2003): 791-801; P. Sikes, “Methodology, Procedures and Ethical Concerns,” in C. Opie, ed., *Doing Educational Research* (Los Angeles: Sage, 2004): 15-33.

[15] See, e.g., M.A. Pember , “The Oglala Sioux Tribe Passes Hate Crime Law Protecting Its LGBTQ Citizens,” *Indian Country Today*, September 19, 2019, *available at* <https://indiancountrytoday.com/news/the-oglala-sioux-tribe-passes-hate-crime-law-protecting-its-lgbtq-citizens> (last visited July 26, 2022); S.-a.-d. Edmo and A. Ridings, eds., *Tribal Equity Toolkit 3.0: Tribal Resolutions and Codes to Support Two Spirit and LGBTQ Justice in Indian Country* (2017).

[16] S. Simmons-Duffin, “Transgender Health Protections Reversed By Trump Administration,” NPR, June 12, 2020, *available at* <https://www.npr.org/sections/health-shots/2020/06/12/868073068/transgender-health-protections-reversed-by-trump-administration> (last visited July 26, 2022).

[17] See *Bostock v. Clayton Cnty.*, *supra* note 9.

[18] *Gloucester County School Board v. Grimm*, 141 S.Ct. 2878, 2878 (2021).

[19] *Fulton v. City of Philadelphia, Pennsylvania*, 141 S.Ct. 1868, 1868 (2021).

[20] American Civil Liberties Union, “Legislation Affecting LGBTQ Rights Across the Country 2021,” December 17, 2021, *available at* <https://www.aclu.org/legislation-affecting-lgbtq-rights-across-country-2021> (last visited July 26, 2022).

[21] The evolution of marriage equality provides one example of this phenomenon, with state marriage equality laws and court decisions upholding marriage equality under state constitutions leading to social norm change and the invalidation of the federal law that defined marriage as between one man and one woman. See M.J. Higdon , “(In)Formal Marriage Equality,” Fordham Law Review 89 (2020): 1351–1409, at 1356-1365; *Obergefell v. Hodges*, 576 U.S. 644 (2015) (called into question by *Dobbs v. Jackson Women’s Health Organization* __ S.Ct.___ (2022)).

[22] Conversion therapy bans provide one example of where local governments have taken action to protect LGBTQ and T & N people beyond state law, leading to efforts to preempt local authority to enact such laws. Movement Advancement Project, The Power of State Preemption: Preventing Progress and Threatening Equality, (May 2018). For examples of where states have acted to preempt local authority to advance social justice before any local action was even taken. See H. Blair, D. Cooper, J. Wolfe, and J. Worker, *Preempting Progress: State Interference in Local Policy Making Prevents People of Color, Women, and Low-Income Workers from Making Ends Meet in the South*, Economic Policy Institute, Sep. 30, 2020. As noted below, the Arkansas law (prohibiting trans athletes from playing on teams that do not match their genders assigned at birth) discussed in the case study in Section E was adopted even though the lead proponent of the bill was unaware of any transgender athletes playing on school sports teams in the state.

[23] Ark. Code Ann. § 20-9-1501 *et seq.* (West 2022).

[24] A.C.A. § 17-80-501 *et seq.* (West 2022); T. Wang, E. Kelman, and S. Cahill, *What the New Affordable Care Act Nondiscrimination Rule Means for Providers and LGBT Patients*, Fenway Institute (2016), *available at* <https://fenwayhealth.org/wp-content/uploads/HHS-ACA-1557-LGBT-Non-Discimination-Brief.pdf> (last visited July 26, 2022).

[25] *Brandt v. Rutledge*, 551 F. Supp. 3d 882 (E.D. Ark. 2021).

[26] M. Sprayregen, “Support for Transphobic ‘Bathroom Bills’ Is Increasing Amid Nationwide Attack on Trans Rights,” Them, September 30, 2021, *available at* <https://www.them.us/story/transphobic-bathroom-bills-support-increasing> (last visited July 26, 2022).

[27] Tenn. Code Ann. § 68-120-120 (West 2022).

[28] T.C.A. § 49-2-801 *et seq.* (West 2022).

[29] *Bongo Prods*., *LLC v. Lawrence*, 548 F. Supp. 3d 666, 671 (M.D. Tenn. 2021) p. 39.

[30] M. Trimms, Federal Lawsuit Challenging Tennessee’s School Transgender Bathroom Law to be Dismissed as Students Move, *Tennessean*, February 14, 2022, *available at* <https://www.tennessean.com/story/news/politics/2022/02/14/tennessee-school-transgender-bathroom-law-lawsuit-dropped-students-move/6750975001/> (last visited July 26, 2022).

[31] Such boilerplate bills are not uncommon and reflect the immense influence of anti-progressive, anti-LGBTQ+ lobbying groups who disseminate model legislation for states to adopt. See, e.g., D. Avery, “State anti-transgender bills represent coordinated attack, advocates say,” NBC News, February 17, 2021, *available at* <https://www.nbcnews.com/feature/nbc-out/state-anti-transgender-bills-represent-coordinated-attack-advocates-say-n1258124> (last visited July 26, 2022); A. Kotch, “ALEC Leaders Boast About Anti-Abortion, Anti-Trans Bills,” Center for Media & Democracy, October 4, 2021, *available at* <https://www.exposedbycmd.org/2021/10/04/alec-leaders-boast-about-anti-abortion-anti-trans-bills/> (last visited July 26, 2022).

[32] Ala. Code § 16-1-52 (2022); A.C.A. §§ 6-1-107 *et seq*. & 16-130-101 *et seq.* (West 2022); Miss. Code Ann. § 37-97-1 *et seq.* (West 2022); Mont. Code Ann. § 20-7-1306 (West 2022); T.C.A. § 49-6-310 (West 2022); Tex. Educ. Code Ann. § 33.0834 (West 2022); W. Va. Code Ann. § 18-2-25d *et seq.* (West 2022).

[33] See American Civil Liberties Union, *supra* note 20.

[34] A.C.A. §§ 17-80-501 *et seq.* & 20-9-1501 *et seq.* (West 2022).

[35] Wash. Rev. Code Ann. § 49.60.178 (West 2022); R.C.W.A. § 48.43.0128 (West 2022).

[36] R.C.W.A., *supra* note 35.

[37] See, e.g., *Two-Spirit*, Indian Health Service, *available at* <https://www.ihs.gov/lgbt/health/twospirit/> (last visited Feb. 13, 2022) (“Though Two-Spirit may now be included in the umbrella of LGBTQ, [t]he term “Two-Spirit” does not simply mean someone who is a Native American/Alaska Native and is gay.”); T. Lyons, A. Krüsi, L. Pierre, A. Smith, W. Small, and K. Shannon, “Experiences of Trans Women and Two-Spirit Persons Accessing Women-Specific Health and Housing Services in a Downtown Neighborhood of Vancouver, Canada,” *LGBT Health* 3, no. 5 (2016): 373-378, at 377 (“[T]he unique experiences of two-spirit individuals may have been overlooked and as such future research would benefit from two-spirit specific research.”); K.A. Culhane-Pera, S.L. Pergament, M.Y. Kasouaher, A.M. Pattock, N. Dhore, C.N. Kaigama, M. Alison, M. Scandrett, M.S. Thao, and D.J. Satin, “Diverse community leaders’ perspectives about quality primary healthcare and healthcare measurement: qualitative community-based participatory research,” *International Journal for Equity in Health* 20 (2021): 1-13, at 6 (“‘We do not have real representation.’”); Community-Based Research Centre, “CBRC Responds to the Exclusion of Two-Spirit People from BC’s In Plan Sight Report,” September 8, 2021, *available at* <https://www.cbrc.net/cbrc_responds_to_the_exclusion_of_two-spirit_people> (last visited July 27, 2022) (“[T]he report… failed to acknowledge the unique experiences among Two-Spirit and Indigenous queer and trans folks, who face additional barriers and prejudice due to their sexual orientation, gender identity, or gender expression.”).

[38] Cal. Health & Safety Code §§ 102430 & 103425 *et seq.* (West 2022).

[39] Cal. Health & Safety Code § 102875 (West 2022).

[40] Cal. Health & Safety Code § 102935 *et seq.* (West 2022).

[41] Cal. Health & Safety Code § 103425 *et seq.* (West 2022).

[42] Cal. Health & Safety Code § 102875 (West 2022).

[43] Cal. Health & Safety Code § 102935 *et seq.* (West 2022); D. Anguiano, “California will Track Violent Deaths of LGBTQ+ People in Nationwide First,” *Guardian*, September 23, 2021, *available at* <https://www.theguardian.com/us-news/2021/sep/23/california-lgbtq-deaths-track> (last visited July 28, 2022); R.L. Uyeda, “California will become the First State to Track the Violent Deaths of LGBTQ People,” *The Lily*, September 30, 2021, *available at* <https://www.thelily.com/california-will-become-the-first-state-to-track-the-violent-deaths-of-lgbtq-people/> (last visited July 28, 2022).

[44] See, e.g., R. Nixon , Slow Violence and the Environmentalism of the Poor ( Cambridge: Harvard University Press, 2011): at 2 (“By slow violence I mean a violence that occurs gradually and out of sight, a violence of delayed destruction that is dispersed across time and space, an attritional violence that is typically not viewed as violence at all.”); M. Carlisle, “Anti-Trans Violence and Rhetoric Reached Record Highs Across America in 2021,” *Time*, December 30, 2021, *available at* < https://time.com/6131444/2021-anti-trans-violence/> (last visited July 28, 2022); M. Mollmann, “Anti-Trans Rhetoric Is Fueling a Pandemic of Violence,” Fund for Global Human Rights, December 1, 2021, *available at* < https://globalhumanrights.org/commentary/anti-trans-rhetoric-is-fueling-a-pandemic-of-violence/> (last visited July 28, 2022).

[45] K. Alfonseca, “Young Transgender Athletes Grappling with Anti-Trans Sports Legislation,” ABC News, June 4, 2021, *available at* <https://abcnews.go.com/Sports/young-transgender-athletes-grappling-anti-trans-sports-legislation/story?id=77880331> (last visited July 29, 2022).

[46] See, e.g., F. Newport, “The Impact of Increased Political Polarization,” Gallup, December 5, 2019, *available at* <https://news.gallup.com/opinion/polling-matters/268982/impact-increased-political-polarization.> (last visited July 29, 2022).

[47] H.A. Walter-McCabe and M.K. Kinney , “An Argument for Explicit Public Health Rationale in LGBTQ Antidiscrimination Law as a Tool for Stigma Reduction,” Saint Louis University Journal of Health Law & Policy 13, no. 2 (2020): 147–174.

[48] See Spade, *supra* note 1.

[49] R.A. Wilchins , “A Continuous Nonverbal Communication,” in Genderqueer: Voices from Beyond the Sexual Binary, eds. J. Nestle , C. Howell , and R. Wilchins (Los Angeles: Alyson Books, 2002): 11–17.

[50] See Newport, *supra* note 46.

[51] According to Dean Spade, “‘life chances’ is a phrase that captures the many, many vectors of harm and well-being that are being distributed in ways that I’m concerned about. For example, whether or not fresh groceries are available in your community, whether there’s toxic waste and polluting industry nearby where you live, whether in your whole life you’re likely to have a job that interests you, what level your local schools are funded at, whether someone in your family is dying or suffering from lack of healthcare and you’re carrying around the stresses of that.” Dean Spade, interview by Meaghan Winter, Guernica, March 1, 2011.

[52] See Walter-McCabe and Kinney, *supra* note 47.

[53] Centers for Disease Control and Prevention, “Social Determinants of Health: Know What Affects Health,” *available at* <https://www.cdc.gov/socialdeterminants/about.html> (last visited June 30, 2022).

[54] P.B. Perrin , M.E. Sutter , M.A. Trujillo , R.S. Henry , and M. Pugh, Jr., “The Minority Strengths Model: Development and Initial Path Analytic Validation in Racially/Ethnically Diverse LGBTQ Individuals,” Journal of Clinical Psychology 76, no. 1 (2019): 118–136. 31468539 10.1002/jclp.22850 PMC6908758

[55] L. Mizock , and K.T. Mueser , “Employment, Mental Health, Internalized Stigma, and Coping with Transphobia among Transgender Individuals,” Psychology of Sexual Orientation and Gender Diversity 1, no. 2 (2014): 146–158.

[56] K.A. McLemore , “A Minority Stress Perspective on Transgender Individuals’ Experiences with Misgendering,” Stigma and Health 3, no. 1 (2018): 53–64.

[57] See Perrin et al., *supra* note 54.

[58] The survey was conducted by the Trevor Project, an LGBTQ youth suicide prevention and crisis intervention organization. Participants were 34,759 LGBTQ youth ages 13-24 across the U.S, of which 38% identified as T&N. The Trevor Project, National Survey on LGBTQ Youth Mental Health 2021, Report of Key Findings, (last visited June 30, 2022).

[59] Human Rights Campaign, “Policy and Position Statements on Conversion Therapy,” *available at* <https://www.hrc.org/resources/policy-and-position-statements-on-conversion-therapy> (last visited June 30, 2022).

[60] A.H. Grossman , J.Y. Park , and S.T. Russell , “Transgender Youth and Suicidal Behaviors: Applying the Interpersonal Psychological Theory of Suicide,” Journal of Gay & Lesbian Mental Health 20, no. 4 (2016): 329–349. 28344728 10.1080/19359705.2016.1207581 PMC5363722

[61] J.M. Grant , L.A. Mottet , J. Tanis , J. Harrison , J.L. Herman , and M. Keisling , Injustice at Every Turn: A Report of the National Transgender Discrimination Survey, Report of Key Findings, (2011).

[62] A.E. Dastagir , “The Imaginary Predator in America’s Transgender Bathroom War,” *USA Today*, April 28, 2016, *available at* <http://www.usatoday.com/story/news/nation/2016/04/28/transgender-bathroom-bills-discrimination/32594395/> (last visited July 29, 2022).

[63] K. Bender-Baird , “Peeing under Surveillance: Bathrooms, Gender Policing, and Hate Violence,” Gender, Place & Culture 23, no. 7 (2015): 983–988, doi:10.1080/0966369X.2015.1073699; S.L. Cavanagh, *Queering Bathrooms: Gender, Sexuality, and the Hygienic Imagination* (University of Toronto Press, 2010).

[64] J.L. Herman , “Gendered Restrooms and Minority Stress: The Public Regulation of Gender and its Impact on Transgender People’s Lives,” Journal of Public Management & Social Policy 19, no. 1 (2013): 65–80.

[65] The USTS is the largest anonymous survey of T&N adults (N=27,715) for the purpose of exploring gendered experiences across personal, professional, and health domains. S.E. James , J.L. Herman , S. Rankin , M. Keisling , L. Mottet , and M. Anafi , The Report of the 2015 U.S. Transgender Survey (Washington, DC: National Center for Transgender Equality, 2016): at 19.

[66] L.J. Wernick , A. Kulick , and M. Chin , “Gender Identity Disparities in Bathroom Safety and Wellbeing among High School Students,” Journal of Youth and Adolescence 46, no. 5 (2017): 917–930. 28361196 10.1007/s10964-017-0652-1

[67] K.L. Seelman , “‘Transgender Adults’ Access to College Bathrooms and Housing and the Relationship to Suicidality,” Journal of Homosexuality 63, no. 10 (2016): 1378–1399. 26914181 10.1080/00918369.2016.1157998

[68] Minority stressors are experienced, observed, and anticipated stigma and discrimination about a person’s marginalized identity. See Meyer and Frost, *supra* note 8.

[69] K.L. Nadal , C.N. Whitman , L.S. Davis , T. Erazo , and K.C. Davidoff , “Microaggressions Toward Lesbian, Gay, Bisexual, Transgender, Queer, and Genderqueer People: A Review of the Literature,” The Journal of Sex Research 53, no. 4-5 (2016): 488–508. 26966779 10.1080/00224499.2016.1142495

[70] *Id.*; C.E. Deitz , Sexual Orientation Microaggressions and Psychological Well-Being: A Mediational Model (St. Louis: University of Missouri, 2015); D.W. Sue, C.M. Capodilupo, and A.M.B. Holder, “Racial Microaggressions in the Life Experience of Black Americans,” *Professional Psychology: Research and Practice* 39, no. 3 (2008): 329-336.

[71] B. Mustanski , R. Andrews , and J.A. Puckett , “The Effects of Cumulative Victimization on Mental Health among Lesbian, Gay, Bisexual, and Transgender Adolescents and Young Adults,” American Journal of Public Health 106, no. 3 (2016): 527–533; D.R. Williams, S.A. Mohammed, and A.E. Shields, “Understanding and Effectively Addressing Breast Cancer in African American Women: Unpacking the Social Context,” *Cancer* 122, no. 14 (2016): 2138-2149. 26794175 10.2105/AJPH.2015.302976 PMC4815715

[72] A.T. Geronimus , M.T. Hicken , J.A. Pearson , S.J. Seashols , K.L. Brown , and T.D. Cruz , “Do US Black Women Experience Stress-Related Accelerated Biological Aging?: A Novel Theory and First Population-Based Test of Black-White Differences in Telomere Length,” Human Nature 21, no. 1 (2010): 19–38. 20436780 10.1007/s12110-010-9078-0 PMC2861506

[73] *Id.*; Williams et al., *supra* note 71; Sue et al., *supra* note 70; R.L. Simons, M.K. Lei , S.R. Beach , A.B. Barr , L.G. Simons , F.X. Gibbons , and R.A. Philibert , “Discrimination, Segregation, and Chronic Inflammation: Testing the Weathering Explanation for the Poor Health of Black Americans,” Developmental Psychology 54, no. 10 (2018): 1993–2006; T. Craddock, *Intergenerational Trauma in African and Native American Literatures* (Greenville: East Carolina University, 2014); J.F. Palacios and C.J. Portillo, “Understanding Native Women’s Health: Historical Legacies,” *Journal of Transcultural Nursing* 20, no. 1 (2009): 15-27. 30234347 10.1037/dev0000511 PMC7685230

[74] M.S. Wallien , S.H. Van Goozen , and P.T. Cohen-Kettenis , “Physiological Correlates of Anxiety in Children with Gender Identity Disorder,” European Child & Adolescent Psychiatry 16, no. 5 (2007): 309–315. 17401613 10.1007/s00787-007-0602-7

[75] See Sue et al., *supra* note 70.

[76] See Mustanski et al., *supra* note 71.

[77] L.Z. DuBois , S. Powers , B.G. Everett , and R.-P. Juster , ”Stigma and Diurnal Cortisol Among Transitioning Transgender Men,” Psychoneuroendocrinology 82 (2017): 59–66. 28511045 10.1016/j.psyneuen.2017.05.008

[78] Centers for Disease Control and Prevention, *supra* note 53; L. Kcomt , “Profound Health-Care Discrimination Experienced by Transgender People: Rapid Systematic Review,” Social Work in Health Care 58, no. 2 (2019): 201–219, doi:10.1080/00981389.2018.1532941. 30321122

[79] See Meyer and Frost, *supra* note 8 and Minority stressors, *supra* note 68.

[80] M.L. Hendricks , and R.J. Testa , “A conceptual framework for clinical work with transgender and gender nonconforming clients: An adaptation of the Minority Stress Model, Professional Psychology: Research and Practice, 43, no. 5 (2012): 460–467. doi:10.1037/a0029597.

[81] J.G. Kosciw , C.M. Clark , N.L. Truong , and A.D. Zongrone , The 2019 National School Climate Survey: The Experiences of Lesbian, Gay, Bisexual, Transgender, and Queer Youth in Our Nation’s Schools (New York: GLSEN, 2020); R.B. Toomey, C. Ryan, R.M. Diaz, N.A. Card, and S.T. Russell, “Gender-Nonconforming Lesbian, Gay, Bisexual, and Transgender Youth: School Victimization and Young Adult Psychosocial Adjustment,” *Developmental Psychology* 46, no. 6 (2010): 1580-1589; J. Harrison, J. Grant, and J.L. Herman, “A Gender Not Listed Here: Genderqueers, Gender Rebels, and Otherwise in the National Transgender Discrimination Survey,” *LGBTQ Policy Journal at the Harvard Kennedy School* 2, no. 1 (2012): 13-24. 10.1037/a0020705 20822214

[82] K. Asakura and S.L. Craig , ‘“It gets better”… but how? Exploring Resilience Development in the Accounts of LGBTQ Adults,” Journal of Human Behavior in the Social Environment 24, no. 3 (2014): 253–266.

[83] Kosciw et al., *supra* note 81; M.E. Eisenberg and M.D. Resnick , “Suicidality Among Gay, Lesbian and Bisexual Youth: The Role of Protective Factors,” Journal of Adolescent Health 39, no. 5 (2006): 662–668, doi:10.1016/j.jadohealth.2006.04.024. 17046502

[84] The 2019 National School Climate Survey was distributed by GLSEN to assess the experience of LGBTQ youth (N=16,713) ages 13-21, of which 43% identified as transgender or nonbinary. Kosciw et al., *supra* note 81.

[85] J.E. Schindel , “Gender 101 — Beyond the Binary: Gay-Straight Alliances and Gender Activism,” Sexuality Research & Social Policy 5, no. 2 (2008): 56–70.

[86] See Kosciw et al., *supra* note 81.

[87] *Id*.

[88] R. Giblon and G.R. Bauer , “Health Care Availability, Quality, and Unmet Need: A Comparison of Transgender and Cisgender Residents of Ontario, Canada,” BMC Health Services Research 17 (2017): 283–293, doi:10.1186/s12913-017-2226-z; A. Rodriguez, A. Agardh, and B.O. Asamoah, “Self-Reported Discrimination in Healthcare Settings Based on Recognizability as Transgender: A Cross-Sectional Study Among U.S. Citizens, Archives of Sexual Behavior,” 47, no. 4 (2017): 973-985, doi:10.1007/s10508-017-1028-z. 28420361 PMC5395792

[89] See Kcomt et al., *supra* note 78.

[90] K.L. Nadal , A. Skolnik , and Y. Wong , “Interpersonal and Systemic Microaggressions Toward Transgender People: Implications for Counseling,” Journal of LGBTQ Issues in Counseling 6 (2012): 55–82, doi:10.1080/15538605.2012.648583.

[91] See Kinney, *supra* note 8.

[92] G.R. Bauer , R. Hammond , R. Travers , M. Kaay , K.M. Hohenadel , and M. Boyce , “‘I Don’t Think This is Theoretical; This is our Lives’: How Erasure Impacts Health Care for Transgender People,” *Journal of the Association of Nurses in AIDS Care* 20, no. 5 (2009): 348–361; J. Pyne, “Unsuitable Bodies: Trans People and Cisnormativity in Shelter Services,” *Canadian Social Work Review/Revue canadienne de service social* 28, no. 1 (2011): 129-137. 10.1016/j.jana.2009.07.004 19732694

[93] *Id.*; See James et al., *supra* note 65; See Kcomt, *supra* note 78; S.J. Gridley, J.M. Crouch , Y. Evans , W. Eng , E. Antoon , M. Lyapustina , and C. McCarty , “Youth and Caregiver Perspectives on Barriers to Gender Affirming Health Care for Transgender Youth,” Journal of Adolescent Health, 59, no. 3 (2016): 254–261, doi:10.1016/j.jadohealth.2016.03.017; R.L. Stotzer, P. Silverschanz, and A. Wilson, “Gender Identity and Social Services: Barriers to Care,” *Journal of Social Service Research* 39, no. 1 (2013): 63-77, doi:10.1080/01488376.2011.637858. 27235374

[94] See James et al., *supra* note 65.

[95] See Kinney, *supra* note 8.

[96] E. Coleman et al., “Standards of Care for the Health of Transsexual, Transgender, and Gender-Nonconforming People, Version 7,” International Journal of Transgenderism 13 (2011): 165–232, doi:10.1080/15532739.2011.700873.

[97] See Kcomt, *supra* note 78.

[98] See Harrison et al., *supra* note 81; T.M. Cruz, “Assessing Access to Care for Transgender and Gender Nonconforming People: A Consideration of Diversity in Combating Discrimination,” Social Science & Medicine 110 (2014): 65–73, doi:10.1016/j.socscimed.2014.03.032; J. Lewis, *Resilience Among Transgender Adults who Identify as Genderqueer: Implications for Health and Mental Health Treatment* (Accession No. 57395) (Doctoral dissertation, ProQuest Information & Learning, 2008). 24727533

[99] See Bauer et al., *supra* note 92.

[100] See Kcomt, supra note 78.

[101] E. Lombardi , “Enhancing Transgender Health Care,” American Journal of Public Health 91, no. 6 (2001): 869–872. 11392924 10.2105/ajph.91.6.869 PMC1446458

[102] See Bauer et al., *supra* note 92.

[103] S.K. Kattari , B. Atteberry-Ash , M.K. Kinney , N.E. Walls , and L. Kattari , “One Size Does Not Fit All: Differential Transgender Health Experiences by Gender Identity and Sexual Orientation, Social Work and Health Care,” 58, no. 9 (2019): 899–9017, doi:10.1080/00981389.2019.1677279. 31618117

[104] American Academy of Pediatrics, “AAP Continues to Support Care of Transgender Youths as More States Push Restrictions, January 6, 2022, *available at* <https://publications.aap.org/aapnews/news/19021> (last visited July 29, 2022).

[105] A.S. Wyckoff, “AAP Continues to Support Care of Transgender Youths as More States Push Restrictions,” American Academy of Pediatrics, January 6, 2022, *available at* <https://publications.aap.org/aapnews/news/19021> (last visited July 29, 2022).

[106] American Medical Association, “AMA to States: Stop Interfering in Health Care of Transgender Children,” April 26, 2021, *available at* <https://www.ama-assn.org/press-center/press-releases/ama-states-stop-interfering-health-care-transgender-children> (last visited July 29, 2022).

[107] L.D. Hughes , K.E. Gamarel , W.M. King , T. Goldenberg , J. Jaccard , and A.T. Geronimus , “State-Level Policy Stigma and Non-Prescribed Hormones Use among Trans Populations in the United States: A Mediational Analysis of Insurance and Anticipated Stigma,” Annals of Behavioral Medicine 56, no. 6 (2021): 592–604. 10.1093/abm/kaab063 PMC9242548 34390573

[108] B. Link , and M.L. Hatzenbuehler , “Stigma as an Unrecognized Determinant of Population Health: Research and Policy Implications,” Journal of Health Politics, Policy and Law 41 , no. 4 (2016): 653–673 . 27127258 10.1215/03616878-3620869

[109] This principle is what underlies the movement for policies to recognize and take action against racism as a public health crisis, for example. *Id.*

[110] A.K. Perone , “Protecting Health Care for Transgender Older Adults Amidst a Backlash of U.S. Federal Policies,” Journal of Gerontological Social Work 63 (2020): 743–752. 32921277 10.1080/01634372.2020.1808139

[111] See Hughes et al., *supra* note 107.

[112] Wilchins, *supra* note 49.

[113] See Walter-McCabe, *supra* note 47.

[114] See Grant et al., *supra* note 61.

[115] See Link and Hatzenbuehler, *supra* note 108; see Walter-McCabe and Kinney, *supra* note 47.

[116] See Walter-McCabe and Kinney, *supra* note 47.

[117] See, e.g., D. Arnos , E. Kroll , E. Jaromin , H. Daly , and E. Falkenburger , *Tools and Resources for Project-Based Community Advisory Boards, Guidebook,* Oct. 2021.

[118] See, e.g., Y. Asada , A.A. Pipito , J.F. Chriqui , S. Taher , and L.M. Powell , “Oakland’s Sugar-Sweetened Beverage Tax: Honoring the ‘Spirit’ of the Ordinance Toward Equitable Implementation,” Health Equity 5, no. 1 (2021): 35–41; *Healthy Food America, Centering Equity in Sugary Drink Tax Policy: Elements of Equitable Tax Policy Design, Sugary Drink Tax Equity Workgroup Report*, Dec. 2020. 33681687 10.1089/heq.2020.0079 PMC7929915

[119] “Alan Haber of the University of Michigan has warned of the strategy of co-optation which seeks to elevate and ‘buy off’ less militant leadership by giving them status in official advisory or other ‘consensus groups’ dominated by community leaders.” D. Brieland , “Community Advisory Boards and Maximum Feasible Participation,” American Journal of Public Health 61, no. 2 (1971): 292–296. 5541872 10.2105/ajph.61.2.292 PMC1530662

[120] Tokenizing is a collection of problematic treatment of individuals from marginalized populations, including performative inclusion, stereotyping, expecting additional services, and undervaluing their work. For example, tokenizing T&N people can happen when individuals are expected to expend their time and energy to educate others about gender and gender expression without compensation. E. Furman , Bye Bye Binary: Exploring Non-Binary Youths’ Experiences of Mental Health, Discrimination, and Community Belongingness (Canada: Wilfrid Laurier University, 2017).

[121] See Brieland, *supra* note 119.

[122] *Id.* at 296.

[123] In addition to providing compensation for time, CAB members should be reimbursed for costs such as transportation and child care services during meetings to ensure participation. See Brieland, *supra* note 119. Beyond basic reimbursement for time and services, compensation is also an acknowledgment and value of CAB members’ expertise and emotional labor. Providing a healthy meal or snack during meetings can also support participation. S.D. Newman , J.O. Andrews , G.S. Magwood , C. Jenkins , M.J. Cox , and D.C. Williamson , “Community Advisory Boards in Community-Based Participatory Research: A Synthesis of Best Processes,” Preventing Chronic Disease 8, no. 3 (2011): A70. 21477510 PMC3103575

[124] See Brieland, *supra* note 119.

[125] D. Linders , “From E-Government to We-Government: Defining a Typology for Citizen Coproduction in the Age of Social Media,” Government Information Quarterly 29, no. 4 (2012): 446–454.

[126] S.D. Newman , J.O. Andrews , G.S. Magwood , C. Jenkins , M.J. Cox , and D.C. Williamson , “Community Advisory Boards in Community-Based Participatory Research: A Synthesis of Best Processes,” Preventing Chronic Disease: Public Health Research, Practice, and Policy 8, no. 3 (2011): 1–12; N.P. Yuan, B.M. Mayer, L. Joshweseoma, D. Clichee, and N.I. Teufel-Shone, “Development of Guidelines to Improve the Effectiveness of Community Advisory Boards in Health Research,” *Progress Community Health Partnerships* 14, no. 2 (2020): 259-269. 10.1353/cpr.2020.0026 PMC8530020 33416647

[127] See Kinney, *supra* note 8.

[128] See E. Taylor , D. Marino , S. Rasor-Greenhalgh , and S. Hudak , “Navigating Practice and Academic Change in Collaborative Partnership with a Community Advisory Board,” Journal of Allied Health 39, no. 3 (2010): 105E–110E; S. Ortega, M.S. McAlvain, K.J. Briant, S. Hohl, and B. Thompson, “Perspectives of Community Advisory Board Members in a Community-Academic Partnership,” *Journal of Health Care Poor Underserved* 29, no. 4 (2018): 1529-1543.

[129] See, e.g., Ind. H.B. 1041 (2022).

[130] M.K. McGowan , Just Words: On Speech and Hidden Harm (Oxford University Press, 2019).

[131] Wilchins, *supra* note 49.

[132] Content for the transgender equity impact assessment statement was directly informed by the Office of Legislative Oversight, *Racial Equity and Social Justice (RESJ) Impact Statement*, December 6, 2021, *available at* <https://www.montgomerycountymd.gov/OLO/Resources/Files/resjis/2021/Bill43-21RESJ.pdf> (last visited July 29, 2022).

[133] O. Gonzalez, “Indiana Lawmakers Enact Anti-trans Sports Bill after Overriding Veto,” AXIOS, May 24, 2022, *available at* <https://www.axios.com/2022/05/24/indiana-veto-governor-trans-sports> (last visited July 29, 2022).

[134] See DEI tools, *supra* note 12.

[135] Zap is a community organizing tool for gaining attention to a particular topic through clear, concise targeted information, such as infographics and flyers. For example, ACT UP has effectively used zaps for AIDS activism since the 1980s. AIDS Coalition To Unleash Power, “Actions & Zaps,” *available at* <https://actupny.org/documents/newmem2.html> (last visited June 30, 2022). Showing Up for Racial Justice (“SURJ”) holds monthly Zaps on Zoom to keep people up to date on pressing issues. Showing Up for Racial Justice, “Monthly Action Hour Zaps,” *available at* <https://surj.org/monthly-action-hour-zaps/> (last visited June 30, 2022).

[136] A.C.A. § 16-130-101 *et seq.* (West 2022).

[137] C. Frizzell, “Ark. Attorney General Leslie Rutledge pushes bill banning transgender athletes from girls’ school sports,” 5 News Online, February 22, 2021, *available at* <https://www.5newsonline.com/article/news/politics/arkansas-attorney-general-leslie-rutledge-bill-banning-transgender-athletes-from-girls-school-sports/527-37dbc82b-0d30-4268-8699-02f65578d0ac> (last visited July 29, 2022).

[138] A.C.A. § 16-130-101 *et seq.* (West 2022).

[139] A.C.A. § 16-130-101 *et seq.* (West 2022), quoting *United States v. Virginia*, 518 U.S. 515, 553 (1996).

[140] *Id.*

[141] E. Claybrook, “Arkansas Senate to consider G.I.R.L.S. Act, after passes Education Committee,” 40/29 News, March 8, 2021, *available at* <https://www.4029tv.com/article/girls-act-aims-to-ban-transgender-women-from-participating-on-womens-sports-teams-in-arkansas/35592538#> (last visited July 29, 2022).

[142] M.R. Wickline, “Attorney General Leslie Rutledge on top in GOP race for lieutenant governor,” Arkansas Democrat Gazette, May 25, 2022, *available at* <https://www.leslierutledge.com/rutledge-record.html> (last visited July 29, 2022).

[143] M.R. Wickline, “Attorney General Leslie Rutledge on top in GOP race for lieutenant governor,” *Arkansas Democrat Gazette*, May 25, 2022, *available at* <https://www.leslierutledge.com/rutledge-record.html> (last visited July 29, 2022).

[144] Arkansas Senate, “Senator Missy Irvin,” *available at* <https://senate.arkansas.gov/senators/missy-irvin/> (last visited June 19, 2022).

[145] A.C.A. § 16-130-101 *et seq.* (West 2022).

[146] AMAB is short for assigned male at birth, which more precisely and inclusively identifies people and is a better alternative to the policy language “biological males.”

[147] See A.C.A., *supra* note 145.

[148] Similar to note 146, AFAB is short for assigned female at birth.

[149] See A.C.A., *supra* note 145.

[150] *Id.*

[151] We recommend creating a free membership to ResearchGate (www.researchgate.net) to contact authors directly for copies of articles.

[152] See U.N. General Assembly, Universal declaration of human rights (1948).

[153] See James et al., *supra* note 65.

[154] See Office of Legislative Oversight, *supra* note 132; Office of Legislative Oversight, “Racial Equity and Social Justice Impact Statements,” *available at* <https://www.montgomerycountymd.gov/OLO/resjis.html> (last visited June 30, 2022); DEI tools, *supra* note 12.

[155] See Office of Legislative Oversight, *supra* note 132.

[156] For more information about Indigenous assessment tools, see Edmo and Ridings, *supra* note 15; Urban Indian Health Institute, “Indigenous Evaluation,” *available at* <https://www.uihi.org/projects/indigenous-evaluation/> (last visited June 30, 2022).

[157] See Walter-McCabe and Kinney, *supra* note 47.

[158] See Wilchins, *supra* note 49.

[159] See Lavietes, *supra* note 4.

